# Biopolymer Hydrogel Based on Acid Whey and Cellulose Derivatives for Enhancement Water Retention Capacity of Soil and Slow Release of Fertilizers

**DOI:** 10.3390/polym13193274

**Published:** 2021-09-25

**Authors:** Silvie Durpekova, Antonio Di Martino, Miroslava Dusankova, Petra Drohsler, Vladimir Sedlarik

**Affiliations:** Centre of Polymer Systems, University Institute, Tomas Bata University in Zlin, Tr. T. Bati 5678, 760 01 Zlin, Czech Republic; dimartino@utb.cz (A.D.M.); Miroslava.Urbankova@seznam.cz (M.D.); pvalkova@utb.cz (P.D.); sedlarik@utb.cz (V.S.)

**Keywords:** biopolymer hydrogel, acid whey, cellulose derivatives, citric acid, swelling properties, soil improvement

## Abstract

This study describes the development of a renewable and biodegradable biopolymer-based hydrogel for application in agriculture and horticulture as a soil conditioning agent and for release of a nutrient or fertilizer. The novel product is based on a combination of cellulose derivatives (carboxymethylcellulose and hydroxyethylcellulose) cross-linked with citric acid, as tested at various concentrations, with acid whey as a medium for hydrogel synthesis in order to utilize the almost unusable by-product of the dairy industry. The water uptake of the hydrogel was evaluated by swelling tests under variations in pH, temperature and ion concentration. Its swelling capacity, water retention and biodegradability were investigated in soil to simulate real-world conditions, the latter being monitored by the production of carbon dioxide during the biodegradation process by gas chromatography. Changes in the chemical structure and morphology of the hydrogels during biodegradation were assessed using Fourier transform infrared spectroscopy and scanning electron microscopy. The ability of the hydrogel to hold and release fertilizers was studied with urea and KNO_3_ as model substances. The results not only demonstrate the potential of the hydrogel to enhance the quality of soil, but also how acid whey can be employed in the development of a soil conditioning agent and nutrient release products.

## 1. Introduction

The agricultural sector is a major consumer of water, one that utilizes in excess of 70% of the available water resources for growing crops and producing foodstuffs [1]. Recent years have witnessed a scarcity of rainfall, seriously complicating irrigation efforts and highlighting the importance of water management in the future. As a consequence, a great deal of research has been directed at developing superabsorbent hydrogel materials for agricultural use with the aim of ensuring the efficient utilization of water and the sustainability of soil [1,2,3]. Hydrogels intended for agriculture primarily comprise soil conditioners for controlled moistening of the environment, which heightens the water holding capacity of the soil, supply water to plants to encourage growth and serve for the sustained release of fertilizers [3,4]. As a result of the emphasis on environmental protection in recent times, great interest has been shown in developing biodegradable hydrogels based on renewable bioresources (e.g., derivatives from agricultural crops or industrial by-products) [5,6,7]. Such environmentally friendly hydrogels have found various applications, including in agriculture, due to their low-cost, sustainability and biodegradability [6,8].

This study deploys acid whey as a primary component in a hydrogel designed as a soil conditioner capable of the gradual release of a fertilizer, the aim being to effectively utilize a waste product of the dairy industry and reduce associated pollution. Since the hydrogel is based on acid whey, it also has the potential to act as a nutritive agent for boosting the growth, yield and quality of crops due to the high content of organic compounds present in the whey [9].

In this work, a combination of two renewable biopolymers (the cellulose derivatives sodium carboxymethylcellulose (CMCNa) and hydroxyethylcellulose (HEC)) as the form of the polysaccharide/whey-based hydrogel was used. Compared to conventional types originating from acrylate, hydrogels with the CMCNa/HEC binary system demonstrate similar swelling properties and high biodegradability [10,11]. In particular, CMCNa as a polyelectrolyte species, enhances the swelling capacity and sensitivity of a hydrogel to environmental stimuli (i.e., ionic strength and pH) as a consequence of the Gibbs-Donnan effect [11]. However, poor cross-linking efficiency has been reported when only CMCNa is used, stemming from electrostatic repulsion between the charged macromolecules of polyelectrolyte chains which tends to form intramolecular rather than intermolecular crosslinking. For this reason, a mixtures of CMCNa with HEC was used for hydrogel formation, as the introduction of HEC avoids the formation of intramolecular crosslinking, thereby stabilizing the macromolecules in a three-dimensional polymer network [12]. In order to design the fully biodegradable and environmentally friendly hydrogel, a naturally occurring and easily biodegradable citric acid (CA) was applied as a non-toxic alternative to chemical crosslinkers. Hydrogels cross-linked with CA have proven to be stiff enough to maintain their shape and high swelling ratio [10].

This work investigates the effects of different cross-linking concentrations and external stimuli on the swelling behavior of the novel hydrogel. Its water retention and biodegradability in soil are determined, while study of the release of fertilizers (urea and potassium nitrate) from the hydrogel reveals its potential for agricultural and horticultural use.

## 2. Materials and Methods

### 2.1. Materials

Carboxymethylcellulose sodium salt (CMCNa) (Blanose 7HOF, with MW 7 × 10^5^ Da, DS = 0.7, viscosity 1000–2800 cp (1%, 25 °C)) and hydroxyethylcellulose (HEC) (Natrosol 250 HR, with MW 106 Da, MS = 2.5, viscosity 1500–2500 cp (1%, 25 °C)) were purchased from Ashland (Ashland, OH, USA). Acid whey was sourced from Milcom, a.s. (Prague, Czech Republic), as a by-product of the manufacture of cream cheese. Citric acid in anhydrous form (C_6_H_8_O_7_, Sigma-Aldrich, Milano, Italy) was employed for the cross-linking reaction. Urea (CH_4_N_2_O, Lach-Ner, Neratovice, Czech Republic) and potassium nitrate (KNO_3_, Sigma-Aldrich, Milano, Italy) were used for loading capacity of fertilizers and release study.

### 2.2. Preparation of the Hydrogel

Samples were prepared according to a procedure described in Durpekova et al. [13], with minor modification. Briefly, a mixture of the CMCNa and HEC at a weight ratio equal to 3:1 and total polymer concentration of 3 wt% was dissolved in the untreated acid whey under mechanical stirring at room temperature (20–22 °C) for 24 h until a homogeneous solution was obtained. Afterwards, CA at defined concentrations (5, 10 and 15% *w*/*w* of the polymer) was added into the solution to obtain different degrees of cross-linking. The cross-linking reaction was achieved during the following drying at 60 °C for 24 h. The samples prepared in this manner are listed in Table 1.

### 2.3. Hydrogel Characterization

#### 2.3.1. Gel Fraction

To measure the gel fractions of cross-linked hydrogels, the dried hydrogel samples were immersed in distilled water for 48 h, at room temperature until a constant weight was reached. The samples were then dried in vacuum oven (Memmert VO400, Swabach, Germany) at a temperature of 40 °C until it had reached constant weight again. The gel fraction (GF) was calculated ac-cording to the following Equation (1):

(1) $$\mathrm{GF}\;(\%)=\frac{W_{d}}{W_{i}}\;\times \;100$$

where W_i_ is the initial weight of dried sample and W_d_ is the weight of dried gel sample after the extraction with deionized water.

#### 2.3.2. Swelling Ratio (SR) under Various Conditions

The values for SR of the hydrogels were measured in triplicate via the gravimetric method in solutions of different pH (pH 2–10), ionic strength (0.001–1.0 M NaCl) and temperature of the media (10–50 °C). In brief, 3 g of the dried hydrogel was soaked in distilled water for 24 h in the laboratory. The pH of the solutions was adjusted by 1.0 M NaOH and 0.1 M HCl to obtain the given range of pH. The kinetics of swelling were measured by taking the hydrogel out of the solution at defined intervals of time (5, 10, 15, 20, 30, 60, 180, 240 and 1440 min), then excess media was removed with filter paper and the weight recorded. The SR was determined by weighing the samples before and after immersion in distilled water for 24 h using the following Equation (2):

(2) $$\mathrm{SR}\;\left(\%\right)=\left(\frac{W_{s}-W_{d}}{W_{d}}\right)\times 100$$

where W_s_ and W_d_ are the masses of the swollen and dried samples (g), respectively.

#### 2.3.3. Water-Holding Capacity (WHC) in Soil

The efficiency of the prepared hydrogel to retain water in soil was expressed in terms of WHC. In this experiment, the soil was dried at 45 °C until it had reached constant weight. An amount of 1 g of the dried hydrogel (5 mm in diameter) was thoroughly mixed with 50 g of the dried soil and transferred into a plastic pot (W_1_). The bottom of the pot was fitted with a filter paper used for leaking out of un-holding water. Pure soil was used as the control (W_0_). The weight of each pot was measured afterwards, then 50 mL of distilled water was added, and the weight was recorded again (W_2_). The WHC was calculated according to Equation (3) as follows:

(3) $$\mathrm{WHC}\;(\%)=\frac{\left(W_{2}-W_{1}\right)}{W_{0}}\times 100$$

#### 2.3.4. Water-Retention (WR)

The samples from WHC study were then stored at controlled laboratory temperature (22 °C) and the weight monitored daily until no detectable weight loss was observed. Equation (4) below gave values for WR:

(4) $$\mathrm{WR}\;(\%)=\frac{W_{t}-W}{W_{i}-W}\times 100$$

where W is the weight of the sample without water, W_i_ is the weight of the sample after adding the water and W_t_ refers to the weight of the sample after specified time intervals.

#### 2.3.5. Reswelling Capacity (RSC) in Free Water and Soil

The RSC capacity of the hydrogels was evaluated in media and soil. In the former of the two, a defined amount of the hydrogel was swollen in distilled water until equilibrium had been reached, as described above for SR (2.3.1.). The swollen hydrogels were subsequently dried at 40 °C until no variation in weight was observed, upon which they were re-immersed in distilled water until equilibrium was obtained again.

The RSC in soil was investigated as follows. The moist soil samples from WHC study (2.3.2.) were dried at 45 °C to constant weight. After that, 50 mL of distilled water was slowly poured into each pot and the samples were weighed again. This procedure of swelling, drying and swelling was repeated five times for each hydrogel sample to determine the reversibility of the hydrogel and its potential for water reabsorption. The RSC (in percent) of the hydrogel was calculated for each reswelling cycle according to Equation (1) for the SR given above.

#### 2.3.6. Biodegradability of the Hydrogel Using Soil Burial Method

The biodegradability of the hydrogel was studied through burial in garden soil (pH ∼6.0) [14]. Hydrogel samples of defined size (2 × 20 mm^2^) were placed approximately 10 cm beneath the surface of the soil in the pots. Thereafter, 20 mL of water was added to each pot, which was kept at room temperature. Water was supplemented as necessary to replenish the soil samples as they dried through evaporation. The weight of each sample was measured at 5-day intervals. The hydrogel samples were taken out, washed gently to remove the soil from surface and dried at 45 °C. Their extent of degradation was monitored at different stages of biodegradation by visual evaluation, changes in morphology (SEM), chemical structure (FTIR) and calculation of weight loss (WL) according to Equation (5) as follows:

(5) $$\mathrm{WL}\;(\%)=\frac{W_{i}-W_{f}}{W_{i}}\times 100$$

where W_i_ is the initial weight of samples before starting the degradation, whereas W_f_ refers to the weight of the sample after specified time intervals of biodegradation.

#### 2.3.7. Scanning Electron Microscopy

The surface texture of the hydrogel and its inner structure as well as changes in the physical structure of the hydrogel when in soil were investigated using scanning electron microscopy (SEM). Dried hydrogels were mounted on to the base platform for gold coating using vacuum sputter coater in a vacuum of 3 × 10^−1^ atm of argon gas. The coated samples were then analyzed using a scanning electron microscope Nova NanoSEM 450 unit set to an operating voltage of 10 kV.

#### 2.3.8. Attenuated Total Reflectance-Fourier Transform Infrared (ATR-FTIR) Spectroscopy

Infrared spectra for the hydrogel and changes that had occurred in chemical structure through burial in the soil were recorded on a Thermo NICOLET 6700 spectrometer via the ATR technique. The unit was equipped with a diamond crystal and set to the resolution of 2 cm^−1^ and range of absorbance of 400–4000 cm^−1^. At predetermined intervals, the samples were removed from the soil, cleaned and analyzed using FTIR.

#### 2.3.9. GC Analysis of Biodegradation in Soil

A prescribed procedure was adhered to by Sera et al. [15]. In total, 100 mg of dry hydrogel, 5 g of perlite and 5 g of soil of dry weight were weighed out and transferred into 500-milliliter biometric flasks. The flasks were sealed with stoppers equipped with septa and incubated at 25 °C. Headspace gas was sampled at appropriate intervals through the septum with a gas-tight syringe and then injected manually into the GC instrument (GC-2010 Plus, Shimadzu Europa GmbH, Germany), equipped with Porapak Q (1.829 m length, 80/100 MESH) and 5A-molecular-sieve (1.829 m length, 60/80 MESH) packed columns connected in series, as well as a thermal conductivity detector (carrier gas, helium; flow, 53 mL min^−1^; and column temperature, 60 °C). Samples of the gas phase (0.1 mL) were taken and analyzed every week. The concentrations of CO_2_ and O_2_ were derived from the calibration curve obtained, using a calibration gas mixture with declared composition. The percentage of mineralization pertaining to the carbon content of the initial sample was calculated from the CO_2_ concentration found. Endogenous production of CO_2_ by soil in blank incubations was always subtracted to obtain values representing net sample mineralization. Since the blank sample comprised 5 g of soil matter without the presence of a hydrogel sample, any production of CO_2_ related entirely to the soil. From the concentration determined, the value for mineralization (M, in per cent) relating to the initial carbon content of the sample was calculated as follows in Equation (6):

(6) $$M=\frac{m_{\mathrm{gc}}}{m_{s}\;w_{c}}$$

where m_gc_ (mg) is the mass of carbon evolved as CO_2_ and obtained from GC analysis, m_s_ (mg) is the weight of the polymer sample and w_c_ is the percentage (*w*/*w*) of carbon in the material investigated. The value of w_c_ for the given polymer was determined on a total organic carbon (TOC) analyzer (TOC-L, Shimadzu Europa GmbH, Duisburg, Germany), equipped with a solid sample module (SSM-5000A, Shimadzu). Three parallel flasks were run for each sample, along with four blanks.

#### 2.3.10. Loading of the Fertilizers

Two sets of hydrogels were prepared; the first contained a single fertilizer, while the other was a combination at the mass ratio of 1:1. The dried hydrogels were soaked in 200 mL of an aqueous solution containing the fertilizer (0.1 M) for 1, 6, 12 and 24 h to discern maximum adsorption over time. After reaching to the equilibrium the loaded hydrogels were washed with deionized water and dried in vacuum at 40 °C. The amount of fertilizer(s) loaded (L) was obtained using Equation (7):

(7) $$L\;\left(\%\right)=\left(\frac{M_{n}-M_{0}}{M_{0}}\right)\times 100$$

where M_n_ is the weight (in mg) of the previously dried hydrogel after being soaked for time *n* in the solution, while M_0_ is the initial weight of the dried hydrogel.

For the simultaneous loading of KNO_3_ and urea, the same procedure was applied, the only difference being that the previously dried hydrogel was soaked in aqueous solution containing KNO_3_ 0.1 M and urea 0.1 M.

#### 2.3.11. Release Studies in Soil Extract

The individual and simultaneous release of the fertilizers, KNO_3_ and urea from the hydrogel was performed in soil extract. The soil extract was obtained as follows: 20 g of soil was soaked in 1 L of distilled water and placed under an oscillating shaker at 40 °C for 48 h. Afterwards, the solid material was separated out by filtration and the resulting liquid used as the release media. The weight of the dried hydrogel containing the fertilizer(s) was recorded prior to the sample being placed in 200 mL of the extract. At a scheduled time, an aliquot of the media was withdrawn and analyzed for KNO_3_ and/or urea content. The KNO_3_ in the media was determined by variation in the conductivity of the media by a conductivity meter (Gryf 158 HB, Czech Republic), whereas the amount of urea was quantified spectroscopically at 245 nm on a UV spectrophotometer (Cary 300 UVeVis Agilent, Santa Clara, CA, USA) after treatment with 4-(Dimethylamino) benzaldehyde (40 mmol/L).

## 3. Results and Discussion

### 3.1. Swelling Properties

#### 3.1.1. Effect of Temperature, pH and Ionic Strength (External Stimuli) of the Immersed Medium on the Degree of Swelling

One of the key properties for hydrogels designed as a soil conditioner is the capacity to absorb and then hold a high volume of water. The swelling results of the proposed hydrogels based on polysaccharide/whey demonstrate its high swelling capacity (1000–1400%), which is comparable with many cellulose- [10,12,16], starch- [17,18,19] or chitosan [20,21]-based superabsorbent hydrogels obtained with water as the preparation media. Such behaviour can be ascribed to the large amount of water that penetrated and was displaced within the hydrogel matrix as a result of heightened Gibbs free energy in the solution, thereby favouring diffusion [22].

**Figure 1:**
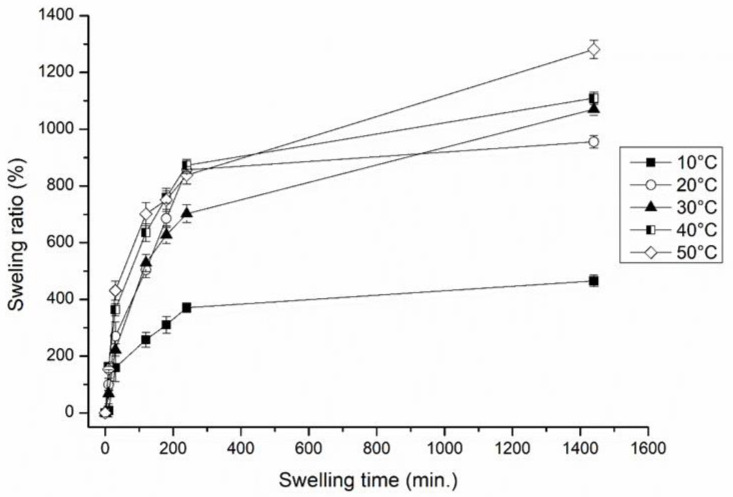
Swelling ratio (%) for hydrogel H5CA immersed in distilled water at different temperatures. The data comprise mean values for SR derived from three instances of water uptake (in weight ± SD; n = 3).

**Figure 2:**
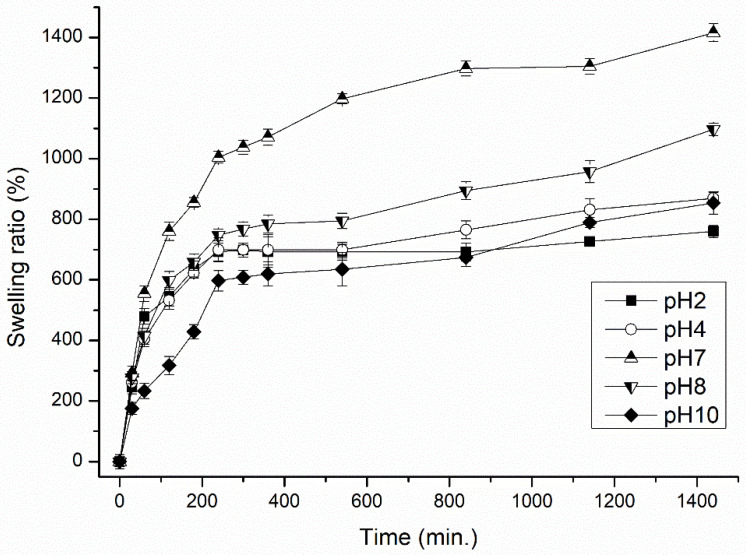
Effect of acidic (pH 2 and 4), neutral (pH 7) and saline (pH 8 and 10) swelling media on swelling behavior (SR %) by hydrogel H5CA within 24 h of swelling. Data refer to the mean value ± SD (n = 3).

**Figure 3:**
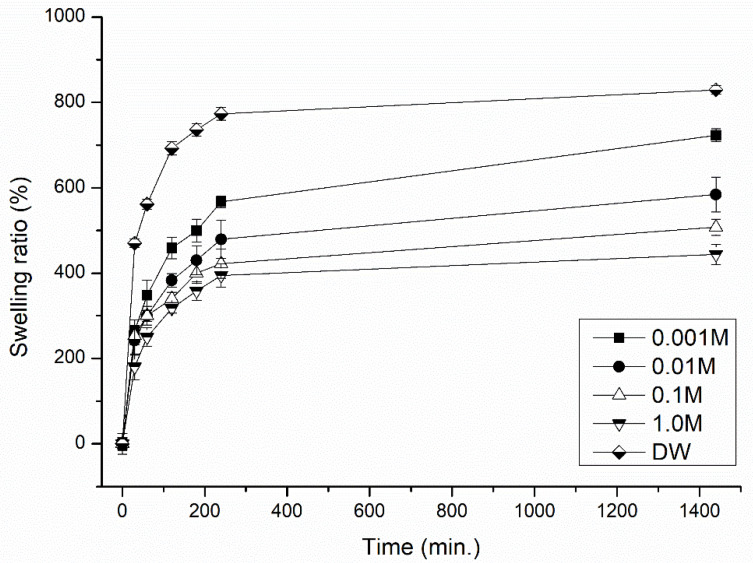
Dependence of ionic strength (concentration of salt) in water on the swelling behavior (SR %) of the hydrogel H5CA. Data refer to mean values ± SD (n = 3).

Swelling kinetics under different conditions of immersed medium was evaluated herein by measuring the water uptake at certain intervals, at pH 2–10, the ionic strength of 0.001–1.0 M NaCl and temperatures of 10–50 °C, so as to determine the effects of such external stimuli on the swelling behavior of the hydrogel samples. Variations in the swelling behavior of hydrogel H5CA are given in Figure 1, Figure 2 and Figure 3. The results reveal that a relationship exists between the temperature and the swelling capacity. As Figure 1 shows, the greatest capacity is evident at higher temperatures, possibly caused by thermal expansion of the hydrogel network and destruction of hydrogen bonding between the polymer molecules; such actions leading to the expansion of the hydrogel matrix after diffusion of the water into the porous structure of the hydrogel [23]. In contrast, the least extent of water uptake was observed when the immersed distilled water was at a low temperature (10 °C); this occurred as a consequence of minimal internal energy and entropy, in turn, leading to limited movement and reduced diffusion of the solution into the internal structure of the hydrogel [24].

Under conditions of low temperature (10 °C), water uptake was slow at the commencement of the swelling process, with the SR stabilizing after 3 h. At a higher temperature (50 °C), however, a significant increase in the SR was observed within the first few hours, followed by a gradual rise. An equilibrium in swelling was reached after 24 h of exposure to the media. Such behavior can be ascribed to the large amount of water that penetrated and was displaced within the hydrogel matrix as a result of heightened Gibbs free energy in the solution, thereby favoring diffusion [22].

The pH of the media and pKa values for the acidic groups in the polymer structure are fundamental to the regulation of swelling properties. A change in the pH of the swelling medium directly impacts the volume of water uptake [25,26]. Swelling media differing from pH were employed to evaluate the impact of pH on the swelling capacity of the polysaccharide/whey-based hydrogel. The graph in Figure 2 shows that a considerable decrease in swelling capacity is evident in the acidic medium at pH 2 and 4. Such be-haviour is associated with the presence of pH-sensitive functional groups in CMCNa, which is the only smart derivative of cellulose with pH-dependent behavior. Under acidic conditions (pH ≤ pKa), the carboxylic groups of CMCNa are protonated in form, causing a reduction in the concentration of the anionic groups and a decrease in swelling. As the pH of the medium exceeds the pKa of the acidic component of the polymer (pH > 4), the carboxylic acid groups become deprotonated and repulsive electrostatic forces between the negatively charged sites (COO^−^) promote chain expansion, facilitating displacement of the molecules of media and enhancing swelling [23,27]. This research indicates that the optimum pH for the swelling of the polysaccharide/whey hydrogel is obtained when the pH of the solution exceeds the pKa of the given functional groups (herein, COOH; pKa 4). Similar results have been reported for pH-sensitive cellulose-based hydrogel composites [20,25,28]. At 8 < pH < 10, swelling capacity reduces through the shielding effect of elevated Na+ concentration in the media which causes inter-anionic repulsive imperfections leading to gradual instability and lacking of the dimensional integrity of the hydrogels in alkaline medium [29].

The hydrogel exhibits the potential for extreme sensitivity in relation to the concentration of ions in the water, as the presence of electrolytes in the solution decreases its capacity for absorption. As detailed in Figure 3, the swelling ratio for H5CA drops when the ionic strength of the solution is high. These findings are in agreement with those reported in the literature [30,31], wherein the swelling capacity of the tested hydrogels demonstrates the same trend through OH^−^ being present in the basic media. In the saline solution, osmotic pressure increases due to the presence of ions, causing desorption of water from the hydrogel [31]; the lowest SR was observed in this medium as a consequence. At low ionic strength, the concentration of charges within the hydrogel network exceeds that of salt in the external solution, and this ion-swelling pressure causes the hydrogel films to expand [32]. The results show that the water absorbency of the polysaccharide/whey hydrogel strongly depends on the salt concentration present in the water. Therefore, it should be noted that the swelling behavior of the hydrogel could alter accordingly in an environment with high ionic strength.

#### 3.1.2. Effect of Concentration of Citric Acid and Degree of Cross-Linking on the Swelling Properties of the Hydrogel

The amount of water that can be held by a sample directly relates to the concentration of the hydrophilic groups and cross-link density [31,33]. During the hydrogel synthesis, at higher temperatures, citric acid esterifies the high hydrophilic carboxylic groups of cellulose derivatives through the formation of a cyclic anhydride intermediate [34]. This reaction leads to new carboxylic acid units, which exhibited to property to forming new intramolecular anhydride groups with an adjacent carboxylic acid unit. The three-dimensional network of the prepared hydrogel is then formed and maintained by cross-linking points [12].

**Figure 4:**
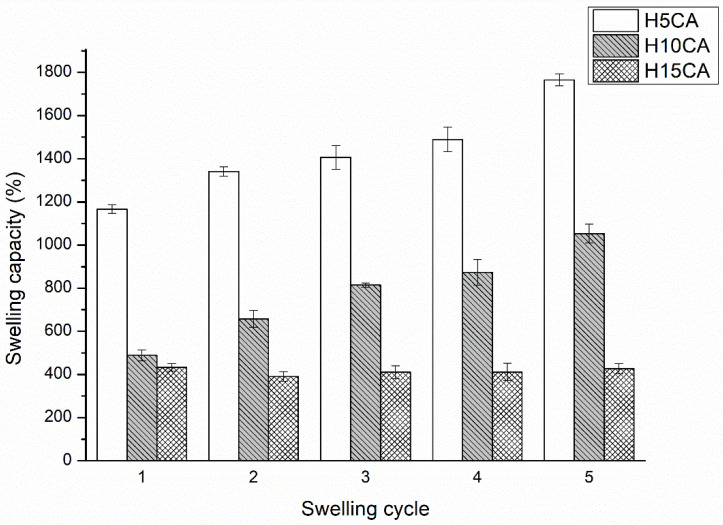
Swelling capacity (%) of the hydrogels H5CA, H10CA and H15CA, following five consecutive re-swelling cycles. Data refer to mean values ± SD (n = 3).

Figure 4 shows the influence of the CA on the water absorbed during the five swelling–drying–swelling cycles. At the highest concentration of CA (15%), less uptake of water was observed, and the swelling capacity was reduced. However, the higher concentrations of CA promoted a rise in carboxyl content and hydrogels of greater structural stability were formed. In contrast, the hydrogel prepared with the lowest concentration of CA was weakly cross-linked, leading to the dissociation of the extremely hydrophilic carboxylic group of CMCNa, thereby exerting an increase in equilibrium swelling and loss of the integrity of the hydrogel. These results clearly demonstrate an inverse correlation between cross-link density and swelling properties. In addition, the results of the gel fraction also confirm the effect of CA on the degree of crosslinking formed in the structure of the hydrogel polymer network and its swelling properties [35]. The more crosslinking that occurs in polymer network, the gel fraction value increases (47.02% ± 2.48% for H5CA, 58.45% ± 1.42% for H10CA, and 67.65% ± 1.52% for H15CA, respectively). The higher concentrations of crosslinking points resulted in lower swelling degree and the hydrogel polymer network became more rigid.

Another observation was that H5CA and H10CA exhibited reversible swelling behavior, with a rise in the swelling capacity during five swelling–drying cycles. This re-swelling ability significantly outperforms that of any cellulosic superabsorbent material reported in the literature [36,37,38], which has shown a rather declining trend with each cycle of swelling–deswelling.

### 3.2. Hydrogel Morphology

**Figure 5:**
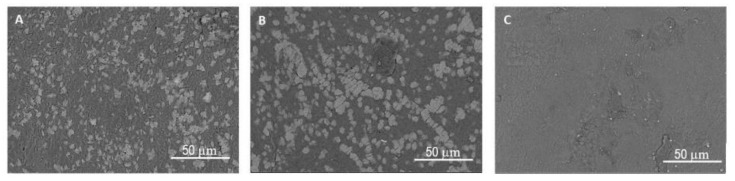
SEM micrographs of the surface of the dried hydrogels with the CA cross-linking agent at the concentrations of (**A**) 5% *w*/*w*; (**B**) 10% *w*/*w* and (**C**) 15% *w*/*w*.

**Figure 6:**
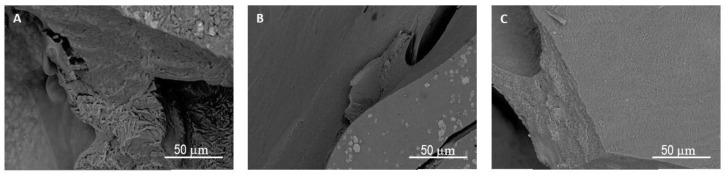
Cross sections of the dried hydrogels with the CA cross-linking agent at the concentrations of (**A**) 5% *w*/*w*; (**B**) 10% *w*/*w* and (**C**) 15% *w*/*w*.

The effects of the concentration of the cross-linking agent on the surface morphology and internal structure of the hydrogel are detailed in Figure 5 and Figure 6, respectively. The micrographs in Figure 5A–C illustrate the surfaces of the hydrogels supplemented with the various concentrations of the cross-linking agent, while Figure 6 shows their cross sections. Unlike surface morphology (Figure 5), which is largely unaffected by the increase in CA content, the inner structure becomes more compact in form. Figure 6A reveals the hydrogel with the lowest concentration of CA (5%) possesses a sponge-like structure, while the higher concentrations are denser in this respect. The inner structures observed correspond with the swelling data. Water uptake is greater and occurs faster in the sponge-like structure, due to the presence of cavities that facilitate the entry and flow of the media.

### 3.3. Water Retention in Soil

**Figure 7:**
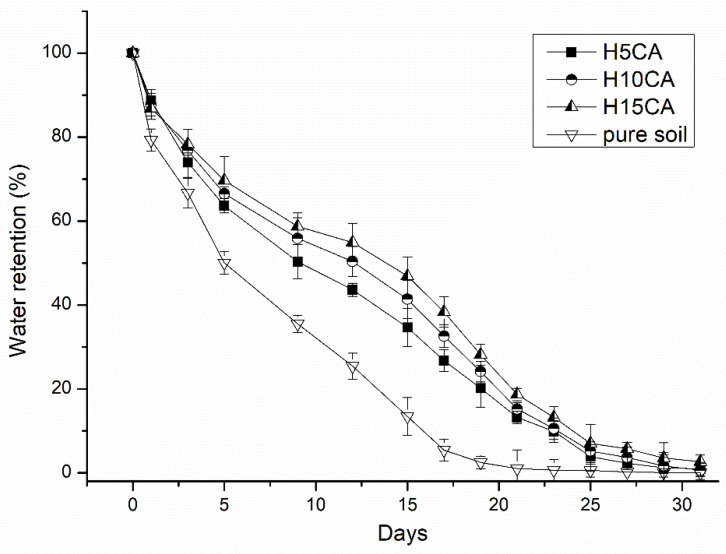
Water retention capacity (%) of pure soil and soil containing the hydrogels H5CA, H10CA and H15CA; data refer to mean values ± SD (n = 3).

Assessing the capacity for water retention in soil is important for understanding of the potential of the hydrogel as a soil conditioner. The graph in Figure 7 shows a continuous decrease in such capacity in soil and loss of water over the course of a month. Water content in the soil, monitored over several days following the first irrigation, was significantly affected by the presence of the hydrogel. The soil samples containing hydrogel possessed substantially more humidity, which confirms their capacity for water uptake. The soil with hydrogel H15CA demonstrated a higher level of water retention exceeding that of pure soil by almost 30%. As the humidity in the soil decreased, the water absorbed by the hydrogel was slowly released, through a mechanism of diffusion [12]. Consequently, agricultural land with soil containing the hydrogel could possess more moisture during periods following irrigation or rain than otherwise, since water would be gradually released from the added material at any times of subsequent dryness [12,39].

### 3.4. Water Re-Absorption in Soil

**Figure 8:**
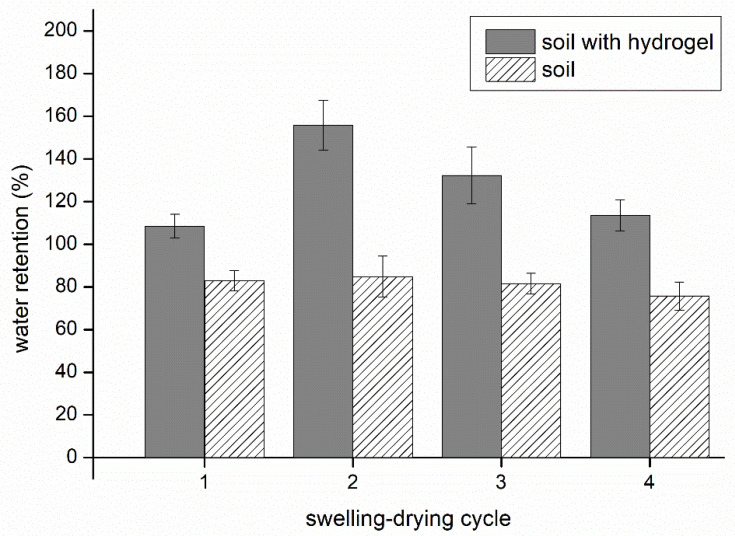
Water retention and re-absorption of hydrogel H5CA in soil during four swelling–drying cycles.

The number of swelling–drying cycles carried out provided information on how reusable the hydrogel was in soil (Figure 8). The samples were gauged for swelling capacity in soil four times; this required that the moist soil was dried to constant weight after each swelling cycle. Each cycle was performed under identical conditions. The results show that the soil amended by the hydrogel was able to repeatedly absorb and retain water after each instance of drying. Adding hydrogel H15CA into the soil resulted in a rise in the absorption capacity compared to pure soil, although variations were observed in water retention values for each swelling–drying cycle. The greatest level of water retention occurred in the second cycle, followed by a gradual drop in the third and fourth. This decrease in the water absorption capacity might have been caused by the hydrogel experiencing degradation and/or variation in its inner structure, as well as change in particle size and ionic osmotic pressure [31]. It should be noted that the swelling degree of the hydrogel could be lower in soil than any values discerned for swelling in distilled water. Since each hydrogel particle in the soil was surrounded by particles of soil under limited pressure from the given environment, water uptake was somewhat diminished.

### 3.5. Release Study

The hydrogel samples were loaded with KNO_3_ and urea, either separately or in combination at the mass ratio of 1:1. The hydrogels soaked in the solutions with a single component reached saturation within different periods-6 h for urea and 1 h for KNO_3_; once saturation had occurred, the given hydrogels were loaded with 72% (0.8 g) of the initial amount of urea and 61% (0.6 g) of KNO_3_. Variations in maximum load and the time in which such loading is achieved are influenced by the chemical structure and solubility of the compounds utilized. Molecular weight and molecular interaction between hydrogel and solute (fertilizer) plays a crucial role in this, since the fertilizers are loaded after preparation of the material, the mechanism for this being diffusion within the hydrogel from the surrounding solution [40]. The swelling of KNO_3_ is controlled by the osmotic pressure of the hydrogel which results from the difference between the mobile ion concentrations between the interior of the hydrogel and external swelling medium [41]. KNO_3_ reached saturation faster than urea, although the final content is affected by the mobility of the ions within the hydrogel structure and solvation. As for the process for the combined load, decrease and inversion in encapsulation efficiency were observed. At equilibrium, only 43% (0.5 g) of urea and 52% (0.5 g) of KNO_3_ were loaded into the hydrogels, potentially as a consequence of equilibrium being affected by the greater volume (200 mL in the combined load vs. 100 mL separately). The differences in cross-link density between the three formulations affected the swelling of the hydrogels, in addition to their release mechanisms and kinetics.

**Figure 9:**
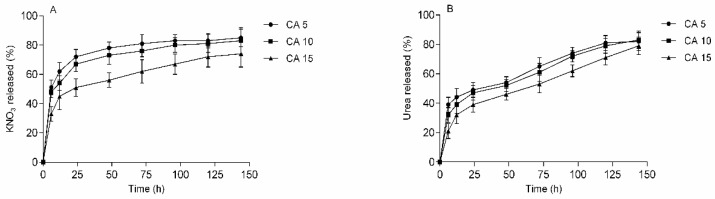
Release of (**A**) KNO3 and (**B**) urea from the hydrogel formulations; the experiments were performed in distilled water at room temperature; data refers to mean values ± SD (n = 3).

The curves in Figure 9 show that release was rapid for both urea and KNO_3_ from the H5CA sample but slower for the H15CA one. All the release profiles demonstrate an intense initial burst that eases off, with over 80% of the loaded compound being released within 5 days.

**Figure 10:**
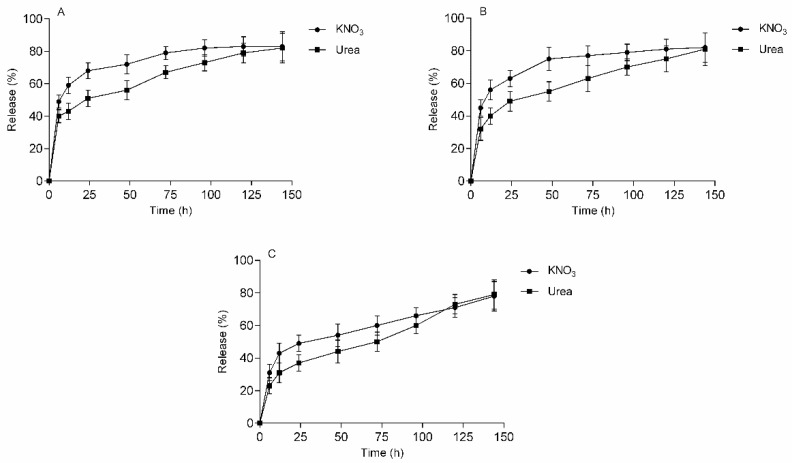
Simultaneous release of potassium nitrate and urea from (**A**) H5CA, (**B**) H10CA and (**C**) H15CA; as performed in distilled water at room temperature; data refer to mean values ± SD (n = 3).

The release profiles for the separate substances (Figure 9A,B) reveal an initial rapid burst took place (Figure 10). The burst peaked at the maximum value for swelling ratio (swelling at equilibrium). This burst release is a result of higher pore size of hydrogel matrix. The electrostatic repulsive effect between the carboxyl groups of hydrogel enhanced the porosity and thus molecules of fertilizer are easily released from the hydrogel matrix [29]. Sample H5CA achieved the highest such burst, while the lowest was seen for H15CA, indicating an inverse correlation with the cross-link density (increasing the concentration of CA resulted in a higher number of cross-linking points and the formation of a more compact microstructure). The samples with urea showed slower release profiles than KNO_3_ due to the occurrence of non-covalent interactions with macromolecular chains, in particular hydrogen bonds between the amino and hydroxyl groups.

No statistical difference existed between the simultaneous release profiles detailed in Figure 10 (*p* < 0.05), suggesting the components neither interacted with each other, e.g., chemically, nor was there any effect on the release or the interaction with the hydrogel matrix. No alterations in the polysaccharide-KNO_3_ or polysaccharide-urea were observed either, due to the simultaneous presence of the fertilizers.

In order to determine the nature of diffusion of fertilizer from hydrogel and relationship with the composition and structure of the hydrogel, the data were processed on the basis of the power of law Equation (8):

(8) $$\frac{M_{t}}{M_{\mathrm{eq}}}=kt^{n}$$

where M_t_ and M_eq_ denote the urea and potassium nitrate diffused from the hydrogel at time t and the point of equilibrium, respectively [42]. The ratio of M_t_ to M_eq_ represents the fertilizer fractional release in time (t), while k is a constant related to the polymer structure. Plotting ln (M_t_/M_eq_) vs. ln t (time) gives coefficient n that describes the type of diffusion which depends upon the interaction in between fertilizer and the components of hydrogel matrix. The result of diffusion coefficient (n), correlation coefficient, R^2^ and release mechanism is given in Table 2. The classification of diffusion mechanism of the fertilizer from the polymeric matrix is as follows: n = 0.5 reveals the Case I or Fickian diffusion (rate of diffusion is much less than the polymer relaxation), n = 1 signifies the case II diffusion (diffusion is greater than time of polymer relaxation) and n between 0.5 and 1.0 signifies the non-Fickian or anomalous diffusion (diffusion and relaxation time is comparable) [4,43]. The n values were found to be 0.66–0.95 indicating the release of fertilizer from the loaded hydrogel samples follows a non-Fickian diffusion and Case II diffusion in case of sample HCA5. 

The data also indicate a direct relationship between the citric acid content and the variation in the release mechanism. Increasing the concentration of CA increases the n value, indicating a reduction of the chain mobility, due to the increase in the concentration of the crosslinking points. This means that a hydrogel with a higher crosslink density shows a more relaxation controlled swelling process [41].

### 3.6. Biodegradation under Aerobic Conditions

Biodegradation is a desirable property for a polymer intended for environmental and agricultural utilization. The course of biodegradation of the hydrogel in soil was quantified herein by detecting and measuring the production of carbon dioxide, through analysis of the gaseous phase using gas chromatography (GC). This provided a higher level of control and objectivity in assessing biodegradation than the usual process of measuring weight loss. The literature describes that the resultant CO_2_ directly relates to the mineralization of the supplied carbon source via bacterial respiration [44]. Aerobic microbes use oxygen as an electron acceptor, breaking down organic chemicals into smaller organic compounds with CO_2_ and by-products of water. Polymer biodegradation proceeds as follows: (i) attachment of the microorganism to the surface of the polymer, whereby extracellular enzymes secreted by the organism cause the primary chain to cleave, leading to the formation of low-molecular-weight fragments, such as oligomers, dimers or monomers (depolymerization); (ii) growth pertaining to the microorganism, biomass uptake and metabolism, whereby low-molecular-weight compounds of the polymer are further utilized by the microbes as a carbon source; and (iii) ultimate degradation of the polymer (biomass + O_2_ → CO_2_ + H_2_O) (mineralization) [45,46]. 

**Figure 11:**
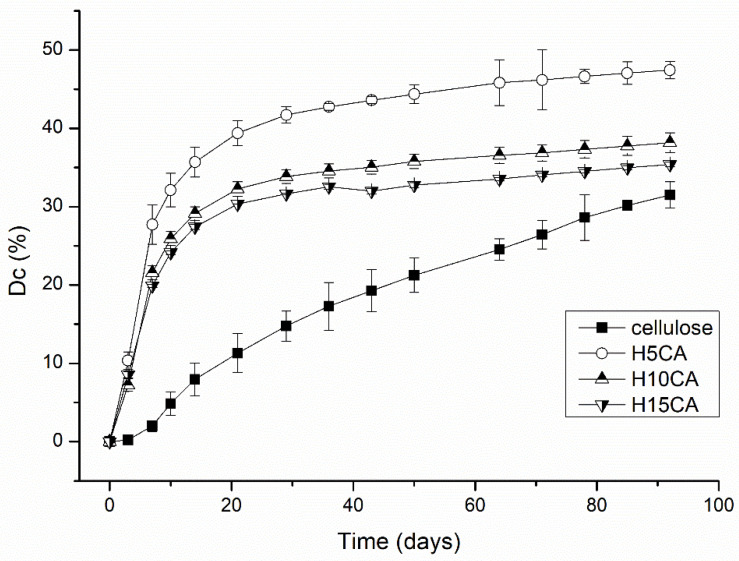
Mineralization curves for the various cross-linked samples, including reference material (cellulose).

Figure 11 plots the mineralization of carbon dioxide of the hydrogel samples during the biodegradation process. They all started to produce significant amounts of carbon dioxide immediately, potentially stemming from the mineralization of the biodegradable whey-polysaccharide material. Notably, the curves for them continued to rise gradually, reaching approximately 30% of mineralization for H15CA and 35% for H10CA, respectively. The level recorded for sample H5CA surpassed this, though, extending to almost 50%, with the curve still displaying a positive trend; this biodegraded more rapidly due to the low cross-linking of the hydrogel structure. Since the material contained whey and derivatives of cellulose, biodegradation of the low cross-linked hydrogel was probably caused by a major increase in the active surface area, resulting in more efficient action by the degrading microorganisms [15]. Another possible factor behind the extent of biodegradation is that the whey could have constituted an additional nutrient for the degrading microorganisms [9].

Based on the results of the soil burial test, it was evident that the compounds forming the hydrogel—whey and cellulose derivatives, were degraded by microorganisms in the soil, despite its inherent cross-linked structure (Table 3).

**Figure 12:**
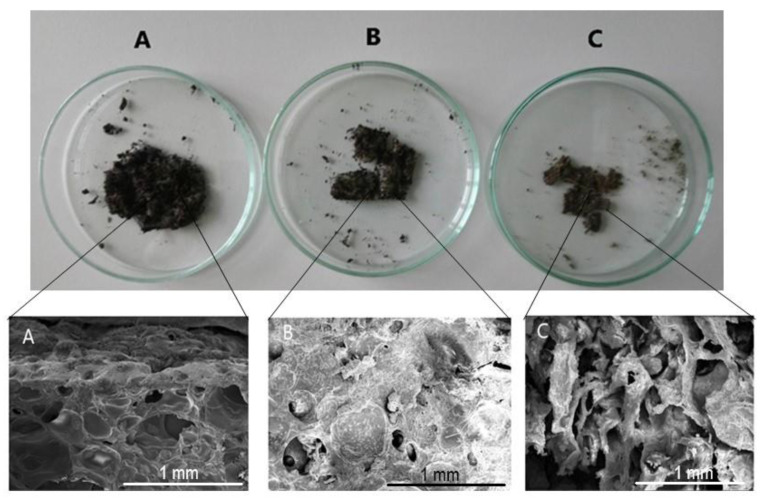
Visual degradation and SEM micrographs of hydrogel H15CA after (**A**) 5 days, (**B**) 10 days and (**C**) 20 days in soil, respectively.

The visual changes the hydrogels underwent during the 20 days of degradation are presented in Figure 12 with H15CA as the example. As is plainly visible in the photographs, the size and shape of the hydrogel changed through exposure to the soil environment over time, undergoing visible reduction in the process. After this period, no further fragmentation was observed. The loss of physical integrity is likely due to the composition of the whey/polysaccharide-based hydrogel, which increases the surface availability for colonization by microorganisms, thereby hastening biodegradation. Therefore, it would be appropriate to enrich such a hydrogel with natural antimicrobial substances that will increase its sustainability in the soil environment.

The SEM micrographs depict the obvious formation of cavities in the inner structure. A stark comparison can be made with the micrograph in Figure 6C of the hydrogel prior to being buried in the soil, revealing that the number and dimensions of the cavities increased over time, causing the structure to collapse after 20 days. 

### 3.7. FTIR Evaluation of Structural Change in the Hydrogel during Biodegradation Process

**Figure 13:**
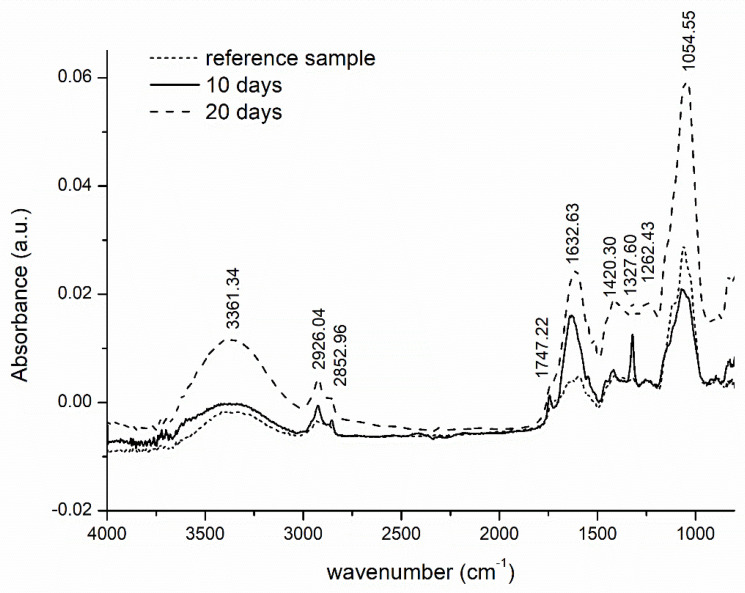
ATR-FTIR spectra for reference sample H15CA, and hydrogel sample after 10 days and 20 days in soil, respectively.

FTIR studies were conducted to determine how the hydrogels changed in the soil environment during the biodegradation test. Figure 13 depicts the FTIR spectra for the hydrogel removed from the soil at certain intervals during the burial experiment.

The reference material (the cross-linked hydrogel of cellulose derivatives and acid whey) shows a broad peak at 3000–3600 cm^−1^, attributed to the stretching vibration of the -OH ascribed to the hydroxyl groups of CA and cellulose derivatives CMC and HEC [10]. The two peaks at 2852 cm^−1^ and the band at 2926 cm^−1^ pertain to C-H stretching of the -CH_2_ and -CH_3_ groups and is characteristic of a cross-linked hydrogel. The peak at 1327 cm^−1^ expresses the presence and bending vibrations of -CH_2_-O-CH groups. Another at 1747 cm^−1^ is attributed to carbonyl (-HC=O) stretching [47]. That at 1632 cm^−1^ denotes the carbonyl band of esters formed during cross-linking and the carbonyl band of free carboxylic acid groups, whose presence is an indication of interaction between the CA and the polymer [48]. The characteristic peak at 1050–1100 cm^−1^ is typical for the absorption bands of CMCNa, related to the C-O stretching vibration of the alcoholic group [10].

The intensity of some peaks for the degraded hydrogel were seen to be lower than those described above, and some had shifted to a different region of the wave number during biodegradation. For example, such a decrease in intensity is evident in the region of 1747 cm^−1^, an expression of ester groups formed by the occurrence of cross-linking with the citric acid. As the samples degrade, the strength of their carboxyl bonds diminishes. The ATR-FTIR spectrum for the hydrogel residues show peaks at 2852, 2400, 2300, 1747 and 1327 cm^−1^, which are reduced in intensity and potentially indicate the loss of functional groups through the microbial breakdown of the samples. It is probable that during biodegradation these cross-linking bonds were the most easily accessible to the enzymes secreted by the microbes, hence they were broken at the beginning of the degradation process. The FTIR spectra also show peaks related to the degradation of cellulose. For example, a new sharp peak at 1547 cm^−1^ is attributed to the presence of free -COOH groups [17], while 1420 cm^−1^ pertains to the stretching vibration of COO and is an indication of the carboxyl groups of CMCNa. The peak at 1262 cm^−1^ denotes the stretching vibration of a new C-C bond from components of the reacted CA and cellulose derivatives. Lastly, a sharp peak appears at 1054 cm^−1^ that is characteristic of the bending vibration of the -OH group.

## 4. Conclusions

This paper describes a set of renewable and biodegradable hydrogels for agricultural use based on acid whey and derivatives of cellulose, prepared as a soil conditioner with the ability to release an added nutrient. The novel hydrogels demonstrate a high capacity for water absorption and properties that mark them out as applicable as carriers of a nutrient or fertilizer. They exhibit pH-dependent, swelling behavior with a swelling ratio exceeding 1400% at the optimal pH and temperature, making them comparable with commercially available products. The novel hydrogels possess good swelling properties in soil and can be reused up to five times. The material is additionally capable of acting as a source of a nutritive whey agents for plants and a means of absorbing and releasing fertilizers in soil. It is also highly biodegradable in soil, since most of its compounds are mineralized when biodegradation commences, indicating its environmentally friendly composition. In summary, acid whey can be successfully used for the preparation of biopolymer biodegradable hydrogels and, thus, reduce the waste of the dairy industry. The novel polysaccharide/whey-based hydrogels are suitable for agricultural application, especially for water retention and nutrient release in soil, and may even have the potential to supersede synthetic, acrylic-based absorbents.

## Figures and Tables

**Table 1:** Composition of the hydrogel samples.

Sample Designation	Cellulose Derivatives (% *w*/*w*)	Citric Acid (% *w*/*w*)
HCA5	3 CMC/HEC (3:1)	5
HCA10	3 CMC/HEC (3:1)	10
HCA15	3 CMC/HEC (3:1)	15

**Table 2:** Release exponent (*n*), correlation coefficient (R^2^) and diffusion mechanism at different hydrogel formulations.

Samples	Fertilizer	*n*	R^2^	Release Mechanism
HCA5	urea	0.92	0.98	Non-Fickian
	KNO_3_	0.95	0.99	Case II diffusion
HCA10	urea	0.66	0.97	Non-Fickian
	KNO_3_	0.81	0.99	Non-Fickian
HCA15	urea	0.69	0.99	Non-Fickian
	KNO_3_	0.73	0.98	Non-Fickian

**Table 3:** Percentage degradation of hydrogels using soil burial method.

Sample Designation	Weight Loss (%) at Different Time Intervals			
	5 Days	10 Days	15 Days	20 Days
H10CA	55.4	67.5	85.5	95.2
H15CA	54.6	62.8	80.8	87.4

## Data Availability

The data presented in this study are available in article.
